# An Inquiry How to Prevent the Small Pox; and Proceedings of a Society for Promoting General Inoculation at Stated Periods, and Preventing the Natural Small Pox, in Chester

**Published:** 1785

**Authors:** 


V. *An Inquiry how to prevent the Small Pox; and Proceedings of a Society for promoting general Inoculation at ſtated Periods, and preventing the natural Small Pox, in Cheſter. By* John Haygarth, M. B. F. R. S. 8vo. *Cheſter,* 1785. 223 pages.

THERE are two opinions, which have ſo generally prevailed as to repreſs every attempt, in this country, to prevent the ſmall-pox; theſe are, 1. That clothes, &c. are rendered dered infectious, by expofure to the variolous effluvia, and, 2. That when the diftemper is epidemical, the whole atmofphere of the place is contaminated. In the work now before us, thefe opinions are very ably and fuccefsfully combated by evidence, which the experience of the Small-pox Society at Chefter, during fix years, has uniformly fupplied. During the whole of this period, we are told, the progrefs of the fmall-pox in that city has been carefully watched, and the caufes of infection invefti-gated with all poffible diligence and attention; but not a fingle fact has occurred to excite a fufpicion that variolous miafms, adhering to clothes, had communicated the diftemper.—Yet, during the whole period, except a few weeks, all the medical practitioners occafionally vifited fmall-pox patients, and the infpectors of the fociety daily entered the infectious chambers: in the fame clothes, without the leaft referve, they approached children liable to infection, but, in no inftance, were known or fufpected to have communicated the diftemper. The Small-pox Society have had, it feems, proofs ftill more numerous and pofitive, that the infectious atmofphere, which furrounds the variolous poifon, is limited within a very fmall

ſmall circumference. The whole experience of the ſociety has uniformly confirmed this opinion; and during the laſt ſix years, we are told, few weeks have elapſed, without ſupplying poſitive proofs that the infectious influence did not extend to the cloſely adjoining houſes.

Dr. Haygarth is of opinion, that the natural ſmall-pox is always communicated through the air; and as he ſuppoſes the mode of combination between the poiſon and the air to be by chemical ſolution, the variolous miaſmata, he thinks, cannot combine themſelves with clothes, &c. unleſs the air is ſuperſaturated with theſe effluvia. This may particularly happen, when clothes, &c. bedaubed with the variolous poiſon, are ſhut up in boxes, or when a letter impregnated with it is folded up, ſo as to exclude all acceſs of freſh air; and our author is inclined to conſider theſe as the moſt uſual methods of transferring the ſmall-pox to a diſtant place.

In an inquiry how to prevent the ſmall-pox, it is of conſequence to determine the periods at which a patient begins and ceaſes to be infectious. Dr. Haygarth relates ſeveral facts, which render it probable that before the eruption appears, the patient is ſeldom or never liable to commucommunicate the diſtemper; and this conjecture receives additional weight from the teſtimony of Dr. Heberden, who, in a letter to the author, has obſerved, that many inſtances have occurred to him, "which ſhew, that one who "never had the ſmall-pox might ſafely aſſociate, "and even lie in the ſame bed with a variolous "patient, for the two or three firſt days of eruption, without receiving the infection."

With reſpect to the time at which a patient ceaſes to be infectious, Dr. Haygarth conjectures it to be after the ſcabs have all dropt off. This conjecture, he ſays, has been confirmed by the uniform experience of the ſmall-pox ſociety. He is aware, however, that the ſkin, hair, &c. may have variolous matter adhering to them, and of courſe render the patient infectious during a longer time, unleſs they be ſufficiently cleaned.

We are indebted to Baron Dimſdale for the very uſeful obſervation *, that the natural infection of the ſmall-pox may be ſuperſeded by inoculation; but the Baron was unable to aſcertain the exact time of receiving the natural in-

* In his Tracts on Inoculation. See the 2d vol. of this Journal, page 164.

fection. From ſome facts related by Dr. Haygarth, it would ſeem that the period between infection and the variolous fever, is about two days longer in the natural than the inoculated infection.

As the infection of the ſmall-pox can only be caught by approaching very near to the variolous poiſon in a recent ſtate, or that has been cloſe ſhut up from the air ever ſince it was recent, and the variolous miaſms do not render clothes, &c. infectious, it follows, that the ſmall-pox may be prevented, by keeping perſons, liable to the diſtemper, from approaching within the infectious diſtance of the variolous poiſon till it can be deſtroyed.

The rules of prevention laid down by the author are ſuch as have been ſucceſsfully adopted by the Small-pox Society at Cheſter, an inſtitution of which we formerly * had occaſion to give ſome account. The proceedings of this Society are given in an appendix to the work, as is likewiſe a very curious letter from Dr. Waterhouſe to the author, deſcribing the regulations by which Rhode Iſland, in America, has hitherto been exempted from the ſmall-pox.

* Vol. 3, page 427.

